# Perceptions of immunization information systems for collecting pandemic H1N1 immunization data within Canada's public health community: A qualitative study

**DOI:** 10.1186/1471-2458-10-523

**Published:** 2010-08-31

**Authors:** Christine L Heidebrecht, Julie Foisy, Jennifer A Pereira, Sherman D Quan, Donald J Willison, Shelley L Deeks, Michael Finkelstein, Natasha S Crowcroft, David L Buckeridge, Maryse Guay, Christopher A Sikora, Jeffrey C Kwong

**Affiliations:** 1 Department of Surveillance and Epidemiology, Ontario Agency for Health Protection and Promotion, Toronto, Canada; 2 University Health Network, Toronto, Canada; 3 Dalla Lana School of Public Health, University of Toronto, Toronto, Canada; 4 Toronto Public Health, Toronto, Canada; 5 Department of Laboratory Medicine and Pathobiology, University of Toronto, Toronto, Canada; 6 Department of Epidemiology, Biostatistics, and Occupational Health, McGill University, Montréal, Canada; 7 Direction de santé publique de Montréal, Vigie et protection, Montréal, Canada; 8 Département des sciences de la santé communautaire, Université de Sherbrooke, Longueuil, Canada; 9 Institut national de santé publique du Québec, Montréal, Canada; 10 Agence de la santé et des services sociaux de la Montérégie, Longueuil, Canada; 11 Centre de recherche de l'Hôpital Charles LeMoyne, Longueuil, Canada; 12 School of Public Health, University of Alberta, Edmonton, Canada; 13 Institute for Clinical Evaluative Sciences, Toronto, Canada; 14 Department of Family and Community Medicine, University of Toronto, Toronto, Canada

## Abstract

**Background:**

Immunization information systems (IISs) are electronic registries used to monitor individual vaccination status and assess vaccine coverage. IISs are currently not widely used across Canada, where health jurisdictions employ a range of approaches to capture influenza immunization information. Conducted in advance of the 2009 H1N1 vaccination campaign, the objectives of this study were to understand the perceived value of individual-level data and IISs for influenza control, identify ideal system functions, and explore barriers to implementation.

**Methods:**

In July and August 2009, semi-structured interviews were conducted with key informants engaged in vaccine delivery and/or pandemic planning at regional, provincial/territorial and federal levels across Canada. Key informants were recruited using a combination of convenience and snowball sampling methodologies. Qualitative analysis was used to extract themes from interview content.

**Results:**

Patient management, assessment of vaccine coverage, and evaluation of safety and effectiveness were identified as public health priorities that would be achieved in a more timely manner, and with greater accuracy, through the use of an IIS. Features described as ideal included system flexibility, rapid data entry, and universality. Financial and human resource constraints as well as coordination between immunization providers were expressed as barriers to implementation.

**Conclusions:**

IISs were perceived as valuable by key informants for strengthening management capacity and improving evaluation of both seasonal and pandemic influenza vaccination campaigns. However, certain implementation restrictions may need to be overcome for these benefits to be achieved.

## Background

Immunization information systems (IISs) are electronic registries containing individual-level vaccination information, usually including additional functionalities such as adverse event reporting, linkage with other electronic registries, or vaccine management [1], and are used to monitor individual vaccination status and assess vaccine coverage. Individual-level data collected in electronic form at the point of influenza immunization provide public health practitioners, planners and clinicians with readily accessible, high-quality information with which to make decisions [2].

User perceptions of electronic patient information systems have been studied broadly [3-7]; however, this research has primarily focused on the experiences and insights of front-line care providers. Vaccination program planners, policy makers and other public health professionals all rely on immunization data, but little is known about these users' perceptions of IISs for influenza vaccination.

Canadian jurisdictions employ a range of approaches to capture seasonal influenza immunization information, including full IISs, physician billing records, paper systems that maintain information in paper format either in aggregate form or at an individual level, and hybrid systems in which paper immunization records are transferred to an electronic database. In advance of the pandemic (H1N1) 2009 influenza vaccination campaign, we were interested in learning about the perspectives of individuals from jurisdictions with access to IISs as well as those from jurisdictions employing less comprehensive data collection methods. In this study we explored the perceptions of key informants from influenza vaccination programs at federal, provincial/territorial and regional levels, and we sought to answer the following questions: (1) What are the perceived benefits of collecting individual-level data, for pandemic, as well as seasonal influenza immunization? (2) Which features and functionalities should an ideal immunization information system encompass? (3) What are the perceived barriers to collecting individual-level data? (4) What are the perceived barriers to achieving an ideal IIS?

## Methods

### Sampling and recruitment

The breadth of information that was sought in this study led us to identify key informants engaged in vaccine delivery and/or pandemic planning in regional, provincial/territorial and federal jurisdictions. To ensure that the findings reflected perspectives from across Canada, recruitment was initiated by approaching members of a national committee representing all thirteen provinces and territories, as well as federal bodies, who are working towards creating a national network of immunization registries. These members were approached electronically, through a recruitment email requesting their participation in a telephone interview. Snowball sampling was used to recruit additional participants by asking interviewees to provide names of other individuals in their jurisdiction involved with pandemic planning, vaccine program development and/or vaccine delivery. Recruitment of participants continued until broad geographic representation had been achieved to the extent possible given the limited availability of our target population during a pandemic period. Ethics approval was granted by the University of Toronto's Health Sciences Research Ethics Board.

### Data collection

A semi-structured approach to data collection was chosen in order to focus each interview session on particular topic areas while providing an opportunity for broader participant insights to be expressed during the course of the dialogue. Key questions were developed by the research team based on study objectives. All interviews were conducted over the telephone by one member of the research team, and recorded with an electronic voice-recorder once consent was obtained. Recordings were transcribed verbatim by an external transcriptionist for analysis purposes.

### Data analysis

Interview data were assessed using conventional content analysis. This approach allows categories and ultimately themes within the data to be discerned [8,9]. Following immersion in the data through reading the transcripts and listening to interview recordings, two members of the research team independently coded interview transcripts. Throughout this process they developed a coding structure, which defined codes and described the relationship between codes and sub-codes when applicable; this structure evolved as necessary to capture new and modified codes. Periodic co-coding, whereby some transcripts were coded by both team members, as well as regular meetings between the coders, ensured continued consistency in creation and application of codes. Approximately 15% of transcripts were co-coded. Disagreement with respect to coding was rare and was resolved through discussion to reach consensus regarding both the definition and scope of the code. Once coding was completed, the codes were imported into qualitative analysis software (QSR NVivo Version 8.0). Informed by the study questions and designed based on trends that were identified through the coding process (an approach both deductive and inductive), a series of queries was created and executed in order to examine, sort, and categorize and the coded text. From these analyses, key themes in the data emerged. Consensus regarding the key themes was reached between the two coders, after which two verification phases were completed: (1) The themes were first reviewed by a team member who had not participated in the coding process but who was familiar with all of the interview transcripts in order to corroborate the findings. (2) Further, observing a process called member checking [10], key informants were asked to review a summary of the themes in order to ascertain the validity of the interpretations. Twelve participants responded to this request, and all indicated that the findings reflected their recollection of the interview content.

## Results

A total of 31 telephone interviews were conducted during July and August 2009. When participants were approached for approval to include their interview data in an analysis for publication, however, five participants declined this request. The findings presented here reflect 26 interviews conducted with 29 participants (three interviews were conducted with more than one individual); 19 of these participants were recruited through snowball sampling. Saturation was reached before the end of the interview process; no new categories or themes were identified after the 13^th ^interview but because of our desire for geographic representation we continued recruitment until this had been achieved.

Respondents included community health nurses, infectious disease coordinators, vaccine program managers, epidemiologists, medical officers of health, and other public health and communicable disease specialists. Participants represented nine provinces and two territories as well as two federal bodies; we were unable to recruit participants from one province and one territory. The majority of participants were involved with pandemic planning, either at a regional, provincial/territorial, or federal level.

4/26 interviews were conducted with users of electronic information systems that captured all vaccinees, and 4/26 systems used by respondents maintained paper records of individual-level data. 6/26 participants' systems retained aggregate counts of vaccinees, and 10/26 systems captured individual-level data electronically for certain sub-populations or in certain jurisdictions. Two respondents had experience with or knowledge about many different approaches to immunization data collection.

### Perceived benefits of collecting individual-level data

Benefits of individual-level influenza immunization data expressed by participants included both observed and envisioned capabilities, depending on whether or not an interviewee had experience capturing electronic data. Most participants perceived IISs to be valuable, while a small number expressed the view that they were of limited use. The benefits of collecting individual-level immunization data electronically that were shared by the largest number of respondents included assessment of vaccine coverage and patient management. These were expressed as important strengths for both seasonal and pandemic influenza vaccination data.

Access to comprehensive vaccination information within an IIS facilitates in-depth evaluation of vaccine coverage and immunization programs overall. Respondents explained that individual-level immunization data allow (or would allow) them to assess progress towards coverage targets and to create a response plan, if necessary, to improve coverage. Further, access to electronic immunization data meant that respondents would be able to engage in more timely analysis and reporting.

I just find it interesting to actually analyze that data and then use it to see what you can do to - as a target, you know, to increase your rates...Are we reaching who we want to and those populations defined at risk?

*In terms of generating a report, you could do it much faster. Because right now we have to physically go through and count all of these things... In terms of the reporting and the records and everything else, it would be a really big time saver*.

The added benefit of being able to look at longitudinal coverage data was also noted.

*We found it very valuable over the years because we've been able to determine trends, who's getting the vaccine and who isn't. And that's allowed us to target specific risk groups where we feel we need to improve coverage*.

Respondents felt that maintaining electronic immunization records allowed them to better manage patient care both at the point of vaccination and during subsequent patient visits. Immunization records that were accessible to other providers were thought to further improve clinical care.

*When there's an immunization registry present, if somebody goes to enter the information, they know that that person's had an adverse event 'cause it's flagged automatically. So they know not to immunize that individual*.

*We had people coming to our health unit saying, "...They won't let me come to work unless I have proof." We would go through boxes of paper consents...and then retrieve that information manually. Now it's just a couple of keyboard clicks and we're there*.

The ability to record and track dose number was identified by a large proportion of respondents as an important feature of IISs during a pandemic immunization campaign. (When the interviews were conducted it was believed that a two-dose schedule would be required for all individuals.) Respondents felt strongly about the need to have accessible information at the point of service to assess a client's prior vaccination history and provide the appropriate service, observing the need to properly space doses in order to ensure effectiveness.

*But in terms of pandemic, because it is the two doses, whatever they determine, 21, you know, 28 days apart, then to ensure that the time period is accurate, that would be important. Because otherwise you're depending on that individual to come back when they're supposed to and, you know, that often doesn't work*.

Other perceived benefits of the collection of individual-level data were expressed by fewer participants. Some respondents described the importance of monitoring adverse events following immunization, especially in the context of a new vaccine containing an adjuvant, and felt that IISs are/would be particularly useful for this, although others noted that it was possible to monitor adverse events without individual-level immunization data. Facilitating the evaluation of vaccine effectiveness through the availability of definitive information about who had been vaccinated and who had not was also expressed as a benefit of an IIS, as was improved efficiency at the point of care.

Two respondents pointed out that there are many unknowns associated with a pandemic - including a new vaccine - and explained that the greater the availability of individual-level data, the more in-depth the analyses that could be conducted when and if needed.

*With pandemic vaccine there's so much that we don't know about it. We don't know will we have a lot of adverse reactions, will we have enough to give two doses immediately, will we have to recall later when we have some more vaccine available... So I feel it's almost more crucial to have the individual information with pandemic than it is with seasonal influenza*.

A small number of participants felt that the increased effort required to collect individual-level data did not outweigh the benefits, and that resources would be more appropriately applied to other elements of the vaccination program. Assuming that individual-level data would have to be collected and captured electronically in two separate phases (which, depending on the type of system, is not necessarily true), they believed that staff time could be better spent engaged in clinical work. Another individual was appreciative of the value of IISs, but cautioned that: "*You have to be careful that, you know, the management of the system doesn't outweigh what you can actually get out of it*."

### Features of an optimal system

It was important to understand which system features would allow key informants to realize the benefits of IISs. Prior to each interview, participants were provided with a proposed set of critical and optional IIS functionalities about which we requested feedback during the interview. Responses described in the following section were based on participants' experiences and supplemented with reactions to this list. Again depending on experience, respondents described features of current systems that were already performing well, or desired characteristics that they perceived as critical to achieving an ideal IIS.

Many key informants described the same several features and functionalities as important for an optimal IIS; system flexibility, rapidity of data capture, and universality were common themes. System portability and widespread accessibility were identified as being key to facilitate consistency of reporting in a range of immunization settings. Web-based systems, as well as systems that could operate in a disconnected mode and subsequently be reintegrated with the central system, were viewed as advantageous for maintaining comprehensive patient registries.

[What] *stands out right away for me is portability, the ability to take that system into remote areas or, you know, school gymnasiums and be able to use that system*.

*The operation in the disconnected mode is essential because, you know, like, when you look at our rural population in *[province], *we do many clinics out in the rural areas and it's really necessary to have that*.

Reporting functionalities were critical to key informants, and many mentioned the value of customizable reporting software. In addition, rapid, efficient data collection and entry was an important consideration for respondents. Features mentioned included pre-populated data fields and bar-code scanning of lot number and client health insurance cards. Lastly, the availability of real-time or close to real-time data was described as ideal by several key informants.

*Obviously it'd have to be easy..., a swipe of the health card, you know, to populate demographics, those kinds of things. I think it's really important that it be quick because where you often get your bottleneck is right at the registration desk... It has to be able to generate reports, those reports that the Ministry requires*.

*We want to be able to look and see how we're doing on an ongoing basis, not just quarterly or whatever... I really think it's important to have real-time connectivity to a central database*.

Many key informants felt that a universal IIS would be of high value both to vaccine administrators as well as vaccinees. Some respondents described the usefulness of a national IIS, while others explained that even a standard system within one health region that was used by all vaccine providers would be an improvement. Universality would allow consistency of immunization records to be maintained to accommodate patients who relocate, and would also permit broader and more comprehensive analyses, including cross-jurisdictional coverage assessment. IISs that were either integrated with or could be linked to other health registries or full electronic health records were mentioned as being particularly ideal.

*Whatever the system is, everybody that has access to the vaccine needs to be reporting in the same way, in real time*.

*People move around and so it would be very helpful to have something that you could really track people, people's immunization no matter where they are and you'd get much better-- higher quality information if you had it all in one place*.

*The biggest shortcoming right now is that our vaccination system is not integrated with the client's health record. That's the number one thing. If we could have all the information we currently collect related to vaccination but have that as a part of the-- one client, one health record concept*.

Additional features that were described by a small number of respondents as being ideal included clinic and inventory management components, reminder tools, flags for missed doses and previous reactions, and the ability to print off immunization records.

### Barriers to implementation

While respondents described many benefits of collecting individual-level immunization data, few public health jurisdictions in the country rely exclusively on IISs for influenza vaccination data collection. Many use IISs or other registries to capture data for certain populations but the capacity of these jurisdictions to assess coverage and conduct other program evaluation exercises is limited. Interview informants consistently described the same key barriers to the implementation and operation of an ideal IIS.

Financial and human resource constraints were identified as barriers by the majority of respondents. Although these constraints were often mentioned generally, many respondents went on to describe system elements that would be particularly resource-intensive. These included the requisite hardware and software, the combination of clinical and technological expertise required to develop and manage an IIS, and the intensity of staff training that would have to be involved to ensure that individuals with clinical responsibilities could interface with the IIS appropriately. The difficulty and expense of ensuring continual remote system access for on-going data collection was also mentioned as a barrier.

*It's hard for me to picture even having that level of information put into an electronic system only because of the amount of PCs that would be needed and the amount of staff training, you know, to get that together. It's a lot easier to have someone trained how to fill in a piece of paper ...and it requires a lot less dollar input*.

*Very few people who know a lot about immunization and a lot about what happens on the front lines and a lot about what analysis could be done, are comfortable enough with the IT components that are required... Similarly, on the IT or the vendor side, they often understand so poorly how immunization works and how the information will be analyzed, they themselves have no idea whether what they're offering makes sense or not. And there's a need to have people who can serve to translate between the two groups and there aren't-- there just aren't very many people like that in the system*.

Some respondents expressed concern about competing financial and political priorities within their jurisdictions. Investing time and resources to develop and implement a new IIS, especially when a national system may be forthcoming, can be difficult to justify. (A new public health system with IIS functionality is being developed in Canada, and while it is anticipated that this system - Panorama - will meet some of the immunization surveillance criteria that are presented here, few details regarding time to implementation are publicly available at the time of this paper's drafting.) Among the participants who felt more strongly about the value of IISs for pandemic influenza than for seasonal, some had trouble with the idea of devoting the resources that would be required toward something that would be most useful for a relatively short period of time.

*I think that the bulk of the funding towards immunization has always been towards purchasing of vaccines and just distributing it. And not a lot of thought's been put to monitoring the records and monitoring coverage. I think that it's just not been a priority. Unfortunately, Panorama, which would have been the answer because it would be a single type of registry that could have been modified across the country for all-- and accessible to all provinces and territories, I'm not sure if it's going to meet all of the requirements*.

*To implement a new system needs a lot of adjustment and a lot of preparation. I think if it's done *[during] *an already difficult situation with lack of resources and extremely high level of emergency, like, pandemic immunization, it adds to the difficulty*...

Patient privacy and confidentiality were identified by several respondents as important issues that must be considered; personal health information legislation - especially when it differs across jurisdictions - may make the implementation of an IIS difficult, or may mean that data may only legally be shared with certain parties. Consent laws in some jurisdictions may limit the accessibility of patient data for linkage or analysis.

*It's certainly the privacy and confidentiality. I hate saying that they're barriers but they-- but it is, in a way...There are criteria that have to be met in order for them to agree to allow us to populate this database that we're trying to develop. So we have to meet those regulations*.

*As it stands we cannot have access to the health insurance database where we would have a common denominator and after it would be easier to put into *[immunization registry], *but it cannot for the time being work this way...Because of individual consent laws. It's very sensitive*.

The last barrier to the implementation of an IIS described by study participants is the fact that in some jurisdictions multiple providers administer influenza vaccines. Key informants pointed out that some providers have no incentive to collect electronic individual-level immunization information, or may not be willing to share their data or to report into a shared electronic system. Even with acceptance by all providers, ensuring that a system is compatible across the full range of health systems in a jurisdiction is challenging.

We are not the biggest provider of flu vaccine in terms of-- who gives the actual immunizations...primary care providers and other agencies are. And they're notoriously bad for giving information...We have many different systems going on here...you have one hospital system, the physicians are probably looking at an electronic record. We're looking at Panorama in public health ...Would there be some way of coordinating so that we'd be one?

## Discussion

In recent years there has been a strong push in Canada for the establishment of electronic health records [11,12], and while the country is moving in the direction of a network of provincial/territorial electronic patient registries, the completion of these registries is likely many years in the future. Findings from this study suggest that the immunization component of these registries will be welcomed by and useful to many public health planners and decision-makers, and that the implementation of systems that permit individual-level influenza immunization information to be captured electronically in the interim would be valuable. Key benefits of collecting data at this level of granularity using an electronic platform as expressed by participants in this study include assessment of vaccine coverage across a range of population groups, care management, and rapid availability of data for reporting and analysis.

There are important distinctions between the collection of individual-level data and a full IIS [1], and in this study we were interested in experiences with and perceptions of both. We observed that the perceived potential usefulness and barriers to implementation vary depending on the approach to data collection that an individual knows of or has experienced. Participants familiar with collection of information on paper followed by manual entry into an electronic system (currently a common approach in Canada) were concerned about how time consuming this process could be and were supportive of system features that would expedite data entry. In contrast, some systems are fully electronic at the point of care and although this implies intensive infrastructure requirements (a computer at every registration and nursing station, for example), it also reduces data entry requirements considerably while providing real-time or close to real-time access to data. Depending on the population size and resources of a particular jurisdiction, the appropriateness of these approaches will vary.

Patient privacy was an important theme that emerged as a barrier to a comprehensive IIS. While it varies by province and territory, privacy legislation in Canada appropriately limits which parties can be custodians of personal health information, and this may mean that individual-level data cannot be shared with particular groups. Further, in order to build a more complete health profile for clients, immunization data from multiple providers must be linked to a common file. To accomplish this, a unique identifier must be sufficiently widely used to be attached to records in multiple environments but adequately flexible to be used across these environments with the appropriate privacy controls. With a growing commitment to the establishment of comprehensive patient registries these are not insurmountable challenges; linkage can be carried out by parties with the authorization to possess personal health information, and de-identified datasets and aggregated data can then be shared among other public health users. When data are required for research purposes, however, navigating multiple jurisdictions' data sharing and approvals processes can prove time-consuming even when patients have given consent for their health information to be used in this way [13].

Lack of common interest in collecting individual-level influenza immunization data and data sharing between public health organizations and other vaccination providers were perhaps the most daunting barriers to the implementation of complete, electronic immunization records revealed by respondents. This is especially challenging in jurisdictions where the majority of vaccinations are administered by community physicians. Participants explained that it is often very difficult to obtain vaccination information from primary care providers (even when this data is recorded electronically), which makes accurate assessment of vaccine coverage nearly impossible. However, these barriers have been overcome in many jurisdictions globally [14-18], as well as within Canada [19,20], through the development of provincial, state and national registries that are designed to receive data from multiple providers. In some areas, financial incentives have been helpful to encourage data sharing; for example, in Australia an 'information incentive' is offered to all vaccine providers who report completed vaccination schedules to the Australian Childhood Immunisation Register [16].

## Limitations

As a result of our sampling approach, the individuals we recruited were almost all representatives of public health organizations. It would be valuable to gain insights into the perspectives of primary care practitioners and other vaccine administrators. Further, because our original sample comprised members of a committee working towards creating a national network of immunization registries, it is not surprising that these individuals were supportive of the collection of individual-level influenza immunization data. Other key informants to whom we were referred by the initial respondents - and who made up the majority of the study population - were not part of this network, however, and thus provided a more balanced perspective. Nevertheless, we acknowledge that this sampling approach may have introduced bias into the work.

This work was conducted between the first and second waves of an influenza pandemic, and results may have been different if we had not interviewed at a time when there was an increased sense of urgency related to immunization data. Likely due in part to the timing of our research, we were unable to recruit participants from two jurisdictions. Respondents from these areas would have added unique and valuable perspective to this work. Further, although they initially consented to contribute to this research as key informants, five individuals from provincial and federal immunization programs declined our request to include their interviews in this analysis. However, other representation from these jurisdictions was included in the analysis.

## Conclusions

Further research is required to evaluate whether there is a link between detailed influenza immunization record-keeping and improved health outcomes. Based on the perceived benefits revealed by this work, however, we recommend that health jurisdictions across Canada consider supporting the implementation of mechanisms to electronically capture individual-level influenza immunization data, and work to improve dialogue between public health and other vaccine providers regarding the collection and sharing of immunization information.

## Abbreviations

IIS: immunization information system

## Competing interests

The authors declare that they have no competing interests.

## Authors' contributions

JF, SQ, MF, NSC, DLB, MG, CAS and JCK designed the study; JF conducted the interviews; and CLH and JAP developed and applied the coding structure, supported by JF, SQ, DJW, SLD, MF, NSC, DLB, MG, CAS and JCK. CLH and JCK drafted the manuscript with contributions from JAP, JF, SQ, DJW, SLD, MF, NSC, DLB, MG and CAS. All authors read and approved the manuscript.

## Pre-publication history

The pre-publication history for this paper can be accessed here:

http://www.biomedcentral.com/1471-2458/10/523/prepub
